# Renoprotective Effects of the Dipeptidyl Peptidase-4 Inhibitor Sitagliptin: A Review in Type 2 Diabetes

**DOI:** 10.1155/2017/5164292

**Published:** 2017-08-27

**Authors:** Cristina Mega, Edite Teixeira-de-Lemos, Rosa Fernandes, Flávio Reis

**Affiliations:** ^1^Agrarian School of Viseu (ESAV), Polytechnic Institute of Viseu (IPV), 3500-606 Viseu, Portugal; ^2^Centre for the Study of Education, Technologies and Health (CI&DETS), Polytechnic Institute of Viseu (IPV), 3500-606 Viseu, Portugal; ^3^Institute of Pharmacology and Experimental Therapeutics and Institute for Biomedical Imaging and Life Sciences (IBILI), Faculty of Medicine, University of Coimbra, 3000-548 Coimbra, Portugal; ^4^CNC.IBILI Research Consortium, University of Coimbra, 3004-504 Coimbra, Portugal

## Abstract

Diabetic nephropathy (DN) is now the single commonest cause of end-stage renal disease (ESRD) worldwide and one of the main causes of death in diabetic patients. It is also acknowledged as an independent risk factor for cardiovascular disease (CVD). Since sitagliptin was approved, many studies have been carried out revealing its ability to not only improve metabolic control but also ameliorate dysfunction in various diabetes-targeted organs, especially the kidney, due to putative underlying cytoprotective properties, namely, its antiapoptotic, antioxidant, anti-inflammatory, and antifibrotic properties. Despite overall recommendations, many patients spend a long time well outside the recommended glycaemic range and, therefore, have an increased risk for developing micro- and macrovascular complications. Currently, it is becoming clearer that type 2 diabetes mellitus (T2DM) management must envision not only the improvement in glycaemic control but also, and particularly, the prevention of pancreatic deterioration and the evolution of complications, such as DN. This review aims to provide an overview of the current knowledge in the field of renoprotective actions of sitagliptin, namely, improvement in diabetic dysmetabolism, hemodynamic factors, renal function, diabetic kidney lesions, and cytoprotective properties.

## 1. Introduction

Type 2 diabetes mellitus (T2DM) is recognized as being a group of chronic diseases characterized by hyperglycaemia where the importance of protecting the body from excessive glucose circulation cannot be overstated. The central key features of T2DM are a defect in insulin resistance and/or insulin secretion, which lead to hyperglycaemia and disrupt the normal relationship between insulin sensitivity and pancreatic *β*-cell function [1]. Degeneration of Langerhans islets with *β*-cell loss is secondary to insulin resistance and is regarded as the most important lesion for disease progression [2–5]. Currently, eight central players are considered to be involved in T2DM pathophysiology—the ominous octet—composed by muscle/liver insulin resistance, *β*-cell failure, enhanced lipolysis, hyperglucagonaemia, dysregulation of hepatic glucose production, brain insulin resistance, increased renal glucose reabsorption, and incretin hormone [glucagon-like peptide 1 (GLP-1) and glucose-dependent insulinotropic polypeptide (GIP)] deficiency, all contributing to a persistent state of hyperglycaemia [6]. GLP-1 and GIP are peptide hormones that are involved in the physiologic regulation of glucose homeostasis. These hormones are secreted from the gastrointestinal tract after a meal and stimulate insulin secretion in a glucose-dependent manner [7]. In T2DM, there is an “incretin defect,” manifested through the reduction in incretin bioavailability, which in part is due to their rapid inactivation by dipeptidyl peptidase-4 (DPP-4) [8]. It is now also acknowledged that biochemical pathways, such as apoptosis, low-grade inflammation, and oxidative stress, which are mainly fuelled by hyperglycaemia and hyperlipidaemia, are key mediators of insulin resistance and *β*-cell dysfunction and are involved in the overall aggravation of the diabetic state [3, 9–15]. The persistent dysfunction of these metabolic pathways in the “ominous octet” organs, through the direct and indirect effects of hyperglycaemia, seems to have an important role in the development of T2DM's major long-term complications [16, 17].

Generally, diabetic complications are divided into macrovascular (coronary artery disease, peripheral arterial disease, and stroke) and microvascular complications (nephropathy, retinopathy, and neuropathy). T2DM-induced micro- and macrovascular complications and their pathologies are major contributors to disease morbidity and mortality, respectively [18, 19]. It is now known that inflammation promotes development and progression of diabetic microangiopathy, which trigger extracellular matrix protein synthesis and capillary basement membrane thickening; these conditions contribute to the development of severe diabetic complications, such as nephropathy, retinopathy, and neuropathy [20–22].

Diabetic nephropathy (DN) originates insidious chronic kidney disease (CKD) and is recognized as the single most common cause of end-stage renal disease (ESRD) and one of the main causes of death in diabetic patients worldwide, being also acknowledged as an independent risk factor for cardiovascular disease (CVD) [23–25].

T2DM can generally be prevented with interventions such as change in dietary habits and physical activity. However, individuals with established diabetes should be treated with antidiabetic drugs [26, 27]. T2DM therapy has vastly improved in the last 10 years with the availability of new drugs and drug classes. These pharmacological agents improve glycaemic control by increasing insulin secretion, ameliorating insulin action, decreasing hepatic gluconeogenesis, and delaying the absorption of carbohydrates [28, 29]. Currently, T2DM can be managed with biguanides, sulfonylureas, meglitinide derivatives, alpha-glucosidase inhibitors, thiazolidinediones, selective sodium-glucose transporter-2 (SGLT2) inhibitors, insulins, amylinomimetics, bile acid sequestrants, dopamine agonists, and incretin-based therapies, which include glucagon-like peptide1 (GLP-1) agonists and DPP-4 inhibitors, of which sitagliptin was the first to be discovered and marketed [29, 30]. Moreover, these drugs may be used in various therapeutic combinations as an add-on therapy for improved management of hyperglycaemia [29].

Treatment regimens of T2DM that reduce the levels of HbA1c to near or below 7% are able to significantly reduce the risk of microvascular complications and diabetes-related death [31–36]. Current recommendations by the consensus of the American Diabetes Association (ADA) and European Association for the Study of Diabetes (EASD) justify the selection of appropriate treatment based on its capability to achieve and maintain desired glycaemic goals [36–38]. Despite all recomendations, many patients spend a long time well outside the target glycaemic range and, therefore, have an increased risk for developing micro- and macrovascular complications [6, 39]. Currently, it is becoming clearer that T2DM management must envision not only glycaemic control but also and particularly, the mechanisms behind progression of pancreatic deterioration and evolution of diabetic complications [40, 41].

The ground-breaking incretin-based therapies that encompass GLP-1 agonists and DPP-4 inhibitors seem to address a previously unmet need in diabetes by modulating glucose supply [42, 43]. In fact, DPP-4 inhibitors, and especially sitagliptin, have progressively increased their therapeutic prominence in the management of T2DM by their capability to potentiate incretin activity. Various studies have described many pleiotropic effects of sitagliptin on various organs and tissues. The knowledge that DPP-4 has the highest expression levels in the kidneys of mammals, which is additionally upregulated in DN [44], indicates that DPP-4 inhibition by sitagliptin is a plausible therapeutic target for management of diabetic nephropathy.

This review outlines the evidence found in previous studies regarding the renoprotective action of sitagliptin in DN, focusing on renal function and lesions, as well as kidney tissue cytoprotective properties, particularly its antiapoptotic, antifibrotic, anti-inflammatory, and antioxidant properties.

## 2. The Incretin System in Diabetic Nephropathy

### 2.1. Overview of DN Pathophysiology

The kidney, besides contributing to the aggravation of hyperglycaemia in T2DM through gluconeogenesis [45] and glucose reabsorption, does not remain unscathed through diabetic evolution, developing progressive lesions and functional impairments that lead to DN [46]. Dysmetabolism, with a central role for chronic hyperglycaemia, and hemodynamic factors, namely, overactivity of the renin-angiotensin-aldosterone system (RAAS) and vascular endothelial growth factor (VEGF) deficiency, have key roles in the pathophysiology of DN. Chronic hyperglycaemia and dyslipidaemia induce mitochondrial deregulation and oxidative stress in kidney cells, which activate several metabolic pathways, including protein kinase C [47], nonenzymatic glycation [48], oxidative stress [49–54], and inflammation [55, 59].

Inflammatory response is mediated by diverse types of inflammatory cells (including macrophages, monocytes, and leukocytes) and molecules (such as adhesion molecules, chemokines, and cytokines, namely, TNF-*α* and IL-1*β*) [55, 60]. Besides altering glomerular hemodynamics and promoting increased vascular permeability, TNF-*α* activates several signalling pathways leading to apoptosis and necrosis. IL-1*β* also modifies vascular permeability and increases the expression of chemokines that induce proliferation and synthesis of extracellular matrix in the mesangium [57]. As inflammation persists, renal tissues are damaged, occurring endothelial dysfunction, mesangial nodule formation (Kimmelstiel-Wilson bodies), renal fibrosis, and apoptosis [55, 60].

Hemodynamic factors [61, 62] predominantly mediated by angiotensin II play a role via overactivity of the RAAS and promotion of VEGF deficiency. Interaction of metabolic factors, such as obesity and chronic hyperglycaemia, alters vasoactive regulating mechanisms of afferent and efferent arteriolar tonus, leading to increased glomerular capillary hydrostatic pressure, hyperperfusion, hyperfiltration, and microalbuminuria. These early renal hemodynamic changes, combined with systemic hypertension, are important in the development and progression of renal disease in T2DM [63].

Albuminuria is mostly glomerular in origin, as albumin must cross the glomerular filtration assembly, which is composed of three main cellular barriers that are of utmost importance for the ultrafiltration process, the fenestrated glomerular endothelial cells, glomerular basement membrane (GBM), and glomerular epithelial cells or podocytes. Alterations in this three-layered structure, like increased intraglomerular pressure, loss of negatively charged glycosaminoglycans in the basement membrane, and further in disease evolution, and increase in basement membrane pore size, contribute to albuminuria [64]. An increasing number of proteins have been identified to be present in foot projections of podocytes. Nephrin is a zipper-like protein that plays a functional role in the structure of the slit diaphragm. The spaces between the teeth of the zipper allow selective transport of small molecules (such as glucose and water) retaining, however, large proteins. Evidence suggests that nephrin could play a key role in glomerular filtration barrier and development of proteinuria as it is found to be downregulated in kidney failure and in diabetic rats [51]. In diabetes, early flattening and retraction of podocytes' foot processes are associated with thickening of the GBM. Thickening of GBM, as well as accumulation of mesangial matrix, and increased numbers of mesangial cells are considered as initial microscopic abnormalities. As the disease progresses, there is a close relationship between mesangial expansion and declining of glomerular filtration. Mesangial expansion also correlates inversely with capillary filtration surface area, which itself correlates to glomerular filtration rate [64]. Long-term persistence of the previous factors ultimately induces histological abnormalities in glomeruli, tubules, interstitium, and renal vascular tissues, affecting basement membranes, podocytes, endothelial, and mesangial cells, which eventually become irreversible [64–68].

The cumulative presence of cooperative risk factors, namely, obesity, hypertension, insulin resistance, hyperglycaemia, dyslipidaemia, and microalbuminuria, appears to support not only the aggravation of CKD but also the development of CVD called the cardiorenal metabolic syndrome [69]. However, the underlying mechanisms of micro- and macrovascular complications of diabetes are not yet completely clarified. It seems that diabetic microangiopathy in conjunction with the aforementioned diabetogenic factors, together with neovascularization of *vasa vasorum,* can lead to macrovascular complications. Consequently, alterations in small arteries and capillaries may be responsible not only for the enduring microvascular complications but also for CVD in diabetes and, thus, may constitute one more link between DN and CVD [18].

### 2.2. The Role of the Incretin System in the Pathophysiology of DN

The presence of the incretin hormone GLP-1 and of its receptor (GLP-1R) in the kidneys suggests that the incretin system can play a role in the modulation of kidney function [70, 71]. Incretin dynamics, which are significantly altered in T2DM, seem also to be implicated in alteration of vascular tonus, natriuretic, and diuretic properties in the kidney [72]. The localization of GLP-1R in endothelial cells and in the proximal renal tubules plays a role in regulating the composition of urine. Stimulation of the GLP-1R in blood vessels results in relaxation of smooth muscle and increased renal blood flow [73].

In the normal kidney, stimulation of GLP-1R by GLP-1 results in inactivation of the Na^+^/H^+^ exchanger isoform 3 (NHE3) transporter, blocking Na^+^ and other electrolytes retrieval from tubular fluid, thus resulting in natriuresis and water loss, and possibly, lowered blood pressure [74]. However, DPP-4 has its highest cellular expression in the kidneys of mammals, being found in the brush border of the proximal tubules, endothelium of the glomerular capillaries, and epithelium of Bowman's capsule [8, 44, 75]. In T2DM, DPP-4 is additionally upregulated in glomeruli of patients with DN, being implicated in the reduction of the half-life of GLP-1 in the kidney [44, 76] and altering its natriuretic and diuretic properties [76].

Other pathophysiological interventions by DPP-4 seem to involve its interaction with extracellular matrix proteins in the kidney during the development and evolution of DN, but there is still insufficient data demonstrating that selective DPP-4 inhibition is able to affect these independent interactions [75]. The association between DPP-4 and integrin *β*1 appears to promote endothelial-to-mesenchymal transition (EndMT) by negatively regulating endothelial viability signalling via suppression of the VEGF-receptor 2 and induction of VEGF-receptor 1 in endothelial cells. It seems that DPP-4 inhibition is capable of inhibiting EndMT and transforming growth factor-*β*2- (TGF-*β*2-) induced Smad3 phosphorylation, and thus, the progression to renal sclerosis. EndMT is a known contributor to the accumulation of activated fibroblasts and myofibroblasts in kidney fibrosis [77].

Furthermore, DPP-4 might be implicated in the inactivation of stromal-derived factor-1 alpha (SDF-1*α*), a chemokine linked to the migration of hematopoietic and endothelial progenitor cells (EPCs) to sites of ischemic injury, involved in tissue repair and in the response to tissue hypoxia [44]. It has been reported that DPP-4 inhibition is able to recruit EPCs to sites of [78].

Direct effects of DPP-4 on immune cells and indirect effects through GLP-1-dependent and GLP-1-independent pathways suggest that enzyme inhibition may have beneficial effects beyond glycaemic control, which may contribute to CKD and CVD outcomes [71].

## 3. Sitagliptin

### 3.1. Pharmacokinetic and Pharmacodynamic Properties of Sitagliptin

Sitagliptin is an oral antidiabetic drug with a recommended dose of 100 mg once a day. Oral absorption is not affected by food. Sitagliptin displays 87% of bioavailability and a reversible fraction bound to plasma proteins of 38% [79]; its half-life is around 12.4 hours; hepatic metabolism of sitagliptin is minimal, mainly by cytochrome P450 3A4, while excretion occurs mainly (70–80%) by the kidney in its unchanged form, with a renal clearance of approximately 350 ml/min [80]. In general, the pharmacokinetic profile of sitagliptin is similar in both healthy volunteers and T2DM patients. The pharmacokinetic properties of the drug have also been evaluated in special patient populations with varying grades of hepatic and renal dysfunction. As a result of its metabolism and elimination route, dose adjustment is only required in patients with severe renal insufficiency, being effective and safe in patients with mild/moderate renal or hepatic impairment [81–85]. No dosage adjustment is necessary related to age, gender and race, or body mass index. Sitagliptin also has a low propensity for pharmacokinetic drug interactions [7].

Sitagliptin is a potent and highly selective DPP-4 competitive inhibitor that does not affect the closely related enzymes DPP-8 or DPP-9 at therapeutic concentrations [75–86]. Sitagliptin acts by inhibiting over 80% of the activity of DPP-4 enzyme (at 12 h postdose for 50 mg/day and at 24 h postdose for ≥100 mg/day), which is responsible for degrading GLP-1, preventing therefore its inactivation. This increases and prolongs plasma concentrations of the active form of GLP-1, allowing the consequent stimulation of insulin synthesis and secretion from pancreatic *β*-cells in a glucose-dependent manner [87–90].

As T2DM patients exhibit relative resistance to the actions of GIP [91], the main goals of DPP-4 inhibitors are to prolong the beneficial effects of endogenous GLP-1 [92] in order to maintain its insulinotropic activity [93]. Glycaemic levels are then further regulated by the resulting higher insulin levels and glucagon suppression from the direct action of GLP-1 on pancreatic *α*-cells [94]. Sitagliptin reduces blood glucose levels, in either the postprandial or the fasting state. It works differently from the previous drugs available for diabetes treatment and is orally active [95, 96].

Clinical trials have demonstrated the efficacy of sitagliptin in terms of improving glycaemic control in T2DM patients, used as either monotherapy, initial combination therapy (usually with a fixed dose combination of sitagliptin/metformin) or add-on therapy to metformin or to other antihyperglycaemic drugs, with or without metformin. Sitagliptin showed efficacy in decreasing HbA1c, fasting plasma glucose (FPG), and postprandial plasma glucose (PPG) levels and also increasing the proportion of patients achieving target HbA1c levels (<7.0%), as shown in several clinical studies [79, 97–100].

### 3.2. Sitagliptin Affords Protection in Organs Targeted by Diabetes

Experimental studies performed in animal models of T2DM that were treated with sitagliptin showed remarkable beneficial effects on glucose and HbA1c levels, an improvement of insulin resistance, together with promotion of weight loss and amelioration of lipid profile [101–110]. Moreover, sitagliptin was able to consistently alleviate oxidative stress and inflammation, which are key players in diabetes pathophysiology and in the development of DN [51, 57, 103, 111].

Sitagliptin promotes a conjoined improvement in dyslipidaemia and hypertension, which are interactive factors for CKD and CVD [104, 107–111]. Sitagliptin attenuates the progress of atherosclerosis in apolipoprotein-E-knockout mice via AMPK- and MAPK-dependent mechanisms [110]. Several reports have corroborated the cardiovascular protective aspects and have also identified cytoprotective properties, such as a decrease in heart oxidative stress, inflammation, and apoptosis [19, 78, 103, 112–118]. Concerning the impact of sitagliptin on lipid profiles in T2DM patients, the majority of studies reported a beneficial effect on triglycerides (TGs), high-density lipoprotein cholesterol (HDL-c), and low-density lipoprotein cholesterol (LDL-c) [119, 120]. DPP-4 inhibition also appears to improve endothelial function in diabetic patients, in both a GLP-1-dependent and GLP-1-independent manner [121, 122]. Furthermore, sitagliptin was able to increase EPC levels in diabetic patients [78].

Our research group has extensively studied the protective effects of sitagliptin on various organs targeted by diabetes, namely, the pancreas, retina, and kidney, in an animal model of T2DM. Sitagliptin was able to prevent the aggravation of both endocrine and exocrine pancreatic histopathological lesions and presented antiapoptotic and anti-inflammatory properties, as well as decreased insulin resistance and pro-proliferative and angiogenic actions [103, 123]. In the retina, sitagliptin treatment prevented changes in the endothelial subcellular distribution of tight junction proteins and improved nitrosative stress and inflammatory and apoptotic states [124]. Later studies in type 1 diabetic rats revealed that sitagliptin could prevent the increase in blood-retinal barrier permeability and decrease the retinal inflammation state and neuronal apoptosis [125]. Our studies in the kidney also revealed protective properties [8, 126]. Other authors have also found diabetic lesion improvement in the pancreas associated to antiapoptotic, pro-proliferative [111, 127–131], and anti-inflammatory properties [132–134].

Besides decreasing insulin resistance [5, 135, 136] and improving hepatic insulin sensitivity, sitagliptin seems also to prevent steatosis [137] through GLP-1R signalling in the liver and reduction of endoplasmic reticulum stress [138]. GLP-1R has been found to be expressed in human hepatocytes [138]. However, other authors failed to detect GLP-1R mRNA transcripts in human, rat, or mouse liver [139]. Antiapoptotic effects on human hepatoma cells by DPP-4 inhibition have also been identified [140].

Treatment of nonobese diabetic mice with sitagliptin not only prevented linoleic acid-induced adipose tissue hypertrophy but also protected against adipose tissue inflammation [131, 137].

In T2DM rats with uncontrolled neuropathy, sitagliptin as add-on to insulin therapy produced neuroprotective effects and ameliorated hyperalgesia, oxidative stress, and inflammation, more than either drug alone [141].

## 4. Sitagliptin Affords Renoprotection in Diabetic Nephropathy

### 4.1. Effects of Sitagliptin on Renal Function

The effects of sitagliptin on DN, using the ZDF rat, noticeably reduced renal dysfunction and injury in this model. In fact, sitagliptin treatment was able to decrease blood urea nitrogen (BUN) levels to values identical to those observed in lean control rats, suggesting an amelioration of renal function [126]. Nevertheless, serum creatinine levels were unchanged between study groups, which are in accordance with others using the ZDF rat as an animal model [106, 142].

Direct vasodilator effects have also been described for DPP-4 inhibitors [143]. In this regard, interactions of angiotensin II and DPP-4/GLP-1 signalling have been proposed as one of the mechanisms for the blood pressure- (BP-) lowering effect of DPP-4 inhibition [144]. Sitagliptin seems to be able to lower BP in a GLP-1-dependent manner through GLP-1R localized in renal endothelial cells and in the proximal renal tubules, which play a role in regulating the composition of urine. DPP-4 inhibition by sitagliptin administration increases GLP-1 availability which stimulates GLP-1R in blood vessels, through the sequential activation of the PKA/LKB1/AMPK*α*/eNOS axis, thus inducing relaxation of smooth muscle and improvement of renal blood flow [143, 145].

There are solid evidences that the proximal tubules play a major role in microalbuminuria in DN, namely, in early stages of the disease [146, 147]. In addition, stimulation of GLP-1R in the proximal tubules results in increased loss of salt, water, and electrolytes in urine. The latter occurs as the GLP-1Rs situated in proximal convoluted tubules of the kidneys are functionally linked to NHE3 transporters. NHE3 promotes recovery of Na^+^ and other electrolytes from the tubular fluid (and thus from urine), thereby returning them into the circulation. Activation of the GLP-1R by GLP-1 results in inactivation of NHE3, which leads to increased Na^+^ loss in urine, consequentially, through osmotic effects, to increased fluid loss, and possibly, to lowered BP [74]. An association of NHE3 with DPP-4 was found in the proximal tubule, which might affect NHE3 surface expression and/or activity [148]. Furthermore, DPP-4 inhibition, in experimental models of obesity and heart failure, was able to upregulate megalin, a receptor that mediates endocytosis of proteins in the proximal tubule [149, 150]. DPP-4 inhibition improved kidney injury and proteinuria in obese rodent models [126, 150–152]. Consistently, Aroor et al. have demonstrated that increased DPP-4 activity, evoked by angiotensin II, suppresses megalin expression in mice, an effect that was partially abolished by using a DPP4 inhibitor [153].

Effects of GLP-1 on lowering BP have been reported in both animal and human studies [154, 155]. The natriuretic and diuretic properties of GLP-1 were proved in infusion studies in a rat model of salt sensitivity by chronic intravenous infusion of GLP-1 [72]. Although glycaemic levels affect renal pathophysiology, the previously mentioned effects of incretin protection appear to be independent of these levels, although the underlying mechanisms still remain to be clarified [156, 157]. Diuretic and natriuretic actions of DPP-4 inhibitors seem to offer renoprotection in the setting of hypertension and other disorders of sodium retention. However, in the case of sitagliptin, available data is not yet sufficient to confirm this protective effect [74, 76].

### 4.2. Effects of Sitagliptin on Renal Lesions

Although DN has been traditionally considered primarily a glomerular disease, it is now widely accepted that the rate of function deterioration correlates best with the degree of renal tubulointerstitial fibrosis. This suggests that although the primary event is a condition marked by glomerular changes resulting in proteinuria, the long-term outcome is determined by events in the renal interstitium [158, 159].

In preclinical studies, initial histopathological observations of DN focused mainly on glomerular lesions, alluding, only briefly, to tubulointerstitial lesions and considering their presence as a secondary lesion of DN [160, 161]. The description of vascular lesions in the kidney was absent in animal model studies and could be scarcely found in a few human DN reports. Thus envisioning evaluation conformity and better correlation between human nephropathy and renal lesions observed in animal models, the international histopathological classification, currently approved for human DN, should be adopted in these studies. This histological classification was established in 2010 and evaluates glomerular, tubulointerstitial, and vascular lesions in a semiquantitative manner, according to their severity and tissue distribution [162].

**Figure 1:**
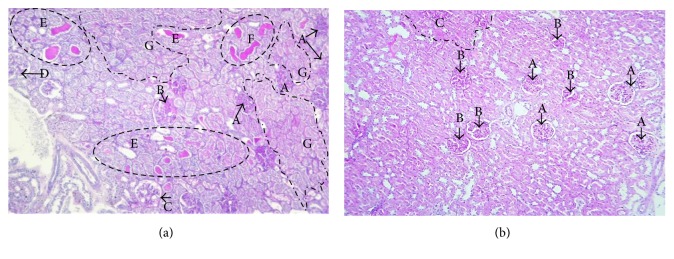
Effects of sitagliptin treatment on diabetic nephropathy lesions in an experimental model of type 2 diabetes. (a) Histopathological lesions in untreated diabetic nephropathy. *Glomerular lesions:* (A) glomerulosclerosis, (B) nodular sclerosis, (C) thickened capsule of Bowman, and (D) normal glomerulus. All other glomeruli on the image display various degrees of mesangial expansion. *Tubulointerstitial lesions:* (E) hyaline cylinders, (F) irregular shape of hyaline cylinders that indicates irregular tubular membranes, (G) various degrees of thickened and irregular tubular basement membranes a characteristic of interstitial fibrosis and tubular atrophy (IFTA). PAS staining of a kidney section from an obese diabetic untreated ZDF rat (original magnification ×100). (b) Improvement of histopathological lesions in sitagliptin-treated diabetic nephropathy*. Glomerular lesions:* Reduction of lesion severity, with global rise in (A) normal glomeruli and (B) the remainder showing various degrees of mesangial expansion, an early lesion of disease. *Tubulointerstitial lesions:* Most of the interstitium has normal appearance, showing only a focal patch of moderate interstitial fibrosis and tubular atrophy (IFTA); PAS staining of a kidney section from an obese diabetic sitagliptin-treated ZDF rat (original magnification ×100).

In experimental animal models, diabetic glomerular lesions initially display thickening of the GBM and mesangial expansion, which are followed by the appearance of nodular sclerosis and vascular pole hyalinization, accompanied by glomerular hypertrophy. With disease aggravation, glomerulosclerosis and glomerular atrophy become evident (Figure 1(a)), confirming the link between diabetes (hyperglycaemia and hyperlipidaemia) and progressive renal injury [8, 126].

In the tubulointerstitium (Figure 1(a)), tubular hypertrophy and associated basement membrane alterations (thickening and irregularity) precede interstitial fibrosis, tubular atrophy (IFTA), and formation of hyaline cylinders, which accompany progressive renal dysfunction (Figure 1(a)). There seems to be a correlation between aggravation of tubulointerstitial and glomerular lesions, which is suggested by the aggravation of both glomeruli and interstitium [126]. Interstitial enlargement also correlates with glomerular filtration, albuminuria, and mesangial expansion. It has been suggested that the accumulation of protein in the cytoplasm of proximal tubular cells causes an inflammatory reaction which leads to tubulointerstitial lesions [64, 163].

**Figure 2:**
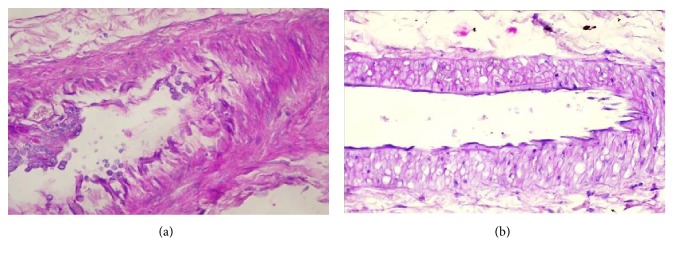
Effects of sitagliptin treatment on diabetic nephropathy vascular lesions in an experimental model of type 2 diabetes. (a) Histopathological lesions in untreated diabetic nephropathy: Renal arteries exhibiting marked hyperplastic arteriosclerosis and thickening and detachment of the intimal layer. Endothelial cells can no longer be identified; PAS staining of a kidney section from an obese diabetic untreated ZDF rat (original magnification ×400). (b) Improvement of histopathological vascular lesions in sitagliptin-treated diabetic nephropathy: A marked reduction in total wall and intimal layer thickening, showing normal endothelial cells; PAS staining of a kidney section from an obese diabetic sitagliptin-treated ZDF rat (original magnification ×400).

Arteriolar hyalinosis and arteriosclerosis are the main vascular lesions found in human DN and also in some experimental animal models of diabetes (Figure 2(a)), and similarly, also aggravate with disease progression [126, 162]. Various studies have shown that DPP-4 inhibition is able to improve renal lesions in experimental animal models. In fact, in the obese diabetic ZDF rat, sitagliptin treatment ameliorated glomerular, tubulointerstitial (Figure 1(b)), and vascular lesions (Figure 2(b)) [126]. Other studies have also reported that suppression of DPP-4 activity and/or protein expression resulted in an amelioration of kidney fibrosis, which was correlated with inhibition of EndMT and reduction of inflammatory and fibrotic markers [164–166]. Similar histopathological improvements with incretin therapies have been disclosed by other studies [166–168].

### 4.3. Renal Cytoprotective Effects of Sitagliptin

Several authors have been postulating that gliptins could theoretically avoid or delay diabetic complications [40, 169–170], namely, due to reduction of oxidative stress and inflammation, as well as by antiapoptotic and pro-proliferative properties on various organs and tissues, including the kidney [101–103, 138, 171, 172].

Considering that sitagliptin is not able to completely normalize hyperglycaemia in studies using low doses [8, 103, 126], an alternative mechanism for the beneficial effect on kidney function/lesions can occur by a direct tissue DPP-4 inhibition, via GLP-1-dependent and/or GLP-1-independent pathways. The GLP-1-dependent activity is reinforced by the expression of GLP-1R in the kidney. In fact, there are several mechanisms by which direct renoprotection could occur. GLP-1 has been associated with the protection of mesangial cells, as well as with the reestablishment of Na^+^, acid-base and fluid homeostasis, which contributes to BP lowering and, collectively, to renoprotection [173–175]. The GLP-1-independent effects have been associated with other known substrates of DPP-4, such as high mobility group box 1 protein (HMGB1), meprin *β*, neuropeptide Y (NPY), and peptide YY (PYY) [76, 164].

It is known that DPP-4 exhibits its enzymatic activity in both membrane-anchored cell-surface peptidase and as a smaller soluble form in blood plasma [77, 131, 176]. In fact, there are some studies suggesting that microvascular endothelial cells are the main sources of endogenous DPP-4 [177, 178]. In addition, in vitro studies showed that both DPP-4 mRNA expression and enzyme activity were enhanced by exposure of human glomerular endothelial cells to high glucose concentrations [179–181]. In agreement, our research group has recently demonstrated that diabetic rats present an increased protein expression of DPP-4 in the kidney, when compared to nondiabetic animals [8].

Experimental studies in ZDF rats showed that chronic hyperglycaemia is associated with increased proinflammatory cytokines, namely, IL-1*β* and TNF-*α* in the kidney [8]. These outcomes are corroborated by other authors that described an increased expression of those proinflammatory cytokines in the diabetic kidney [58, 77, 182], leading to enhanced vascular permeability, oxidative stress, renal hypertrophy, and tubulointerstitial lesions. DPP-4 inhibition by low-dose sitagliptin has prevented the inflammatory profile and the proapoptotic state observed in the diabetic rat kidney, which might justify the improvement in renal function and tissular lesions (glomerular, tubulointerstitial, and vascular lesions). In fact, sitagliptin was able to prevent the increase in both mRNA and protein levels of the proinflammatory cytokines IL-1*β* and TNF-*α* in the diabetic kidneys of ZDF rats [8].

In Wistar rats treated with low- or high-dose sitagliptin during 16 weeks, urinary albumin excretion rate (UAER), serum creatinine, and kidney hypertrophy were significantly decreased. However, creatinine clearance rate and active GLP-1 levels were increased, with more pronounced changes in the high-dose sitagliptin-treated animals. Glomerular lesions were also improved following sitagliptin treatment. Protein and mRNA expression levels of podocalyxin and GLP-1R were significantly increased in both groups, while expression of signal-regulated kinases 1/2 (ERK1/2) and transforming growth factor-*β*1 (TGF-*β*1) was decreased [183]. Podocalyxin is a negatively charged transmembrane glycosaminoglycan that covers the secondary foot processes of the podocytes, which by electrical repellence keeps adjacent foot processes separated, maintaining the urinary filtration barrier open. Podocalyxin depletion is inversely correlated to albuminuria [64, 184]. These overall results also confirm a delay in DN progression promoted by sitagliptin, possibly via the inhibition of ERK1/2 signalling which seems to be activated by AGEs and is implicated in epithelial-myofibroblast transition [185]; by decreased TGF-*β*1 expression, a cytokine associated with inflammatory responses in T2DM, which has been recognized to be involved in the development of glomerulosclerosis and interstitial fibrosis; and by increasing the interaction between GLP-1 and the GLP-1R [186].

Recently, in a study involving 164 DN patients treated with metformin, sitagliptin (100 mg, once a day) was able to decrease UAER, which presented a close correlation with markers of renal fibrosis: TGF-*β*1 and platelet-derived growth factor-BB (PDGF-BB) [187]. Furthermore, PDGF-BB mRNA has been found to be overexpressed in diabetic patients and is considered a factor for mesangial cell proliferation and induction of TGF-*β*1, which shows a profibrotic action, being involved in the development of renal hypertrophy and accumulation of extracellular matrix in DN [188]. In addition, DPP-4 inhibition is known to downregulate TGF-*β*1 expression in mesangial cells [189]. Li et al. [190] suggest that the renoprotective mechanism of sitagliptin may be due to a reduction in protein kinase B (PKB)/Akt levels, which are involved in apoptosis pathways and restoration of adenosine monophosphate-activated protein kinase (AMPK) activity in diverse physiological processes, including ion transport, podocyte function and cell growth and cellular energy homeostasis, inhibition of TGF-*β*1, fibronectin, and p38/ERK MAPK signalling pathways involved in the regulation of ECM expression.

The activation of signalling pathways linked to cell death resulting from chronic hyperglycaemia and to a state of low-grade chronic inflammation contributes to an increase in apoptosis. A proapoptotic state seems to be favoured in the kidney of diabetic ZDF rats, which appears to be mediated by Bax and Bid. Sitagliptin prevented the Bax to Bcl-2 (mRNA and protein) ratio increase and reversed the increase in Bid and TUNEL-positive cells induced by chronic hyperglycaemia in the kidneys of this animal model [8]. In addition, sitagliptin was able to ameliorate serum TG content, thus reducing lipotoxicity-evoked apoptosis in the kidney [8, 190–193].

Additionally, it has been demonstrated that glucose-induced ROS production initiates podocyte apoptosis and its depletion in *vitro* and *in vivo*, leading to DN [49, 56, 194]. Therefore, the reduction of oxidative stress afforded by sitagliptin could eventually reduce ROS production and the consequent risk of cell death. A study on renal ischemia reperfusion damage in diabetic rats found sitagliptin to significantly decrease lipid peroxidation, xanthine oxidase activity, myeloperoxidase activity, and nitric oxide levels in renal tissue in comparison to those in untreated rats. Antioxidant enzymes like glutathione, glutathione peroxidase, superoxide dismutase, and catalase were significantly increased in sitagliptin-treated diabetic rats compared to those in the nontreated ones [195]. Other studies have demonstrated that GLP-1 receptor activation has also attenuated diabetic renal injury by reduction of kidney oxidative stress, inflammation, and apoptosis [196–199].

## 5. Concluding Remarks

The innovative class of DPP-4 inhibitors, such as sitagliptin, seem to address previously unmet needs in diabetes. In fact, DPP-4 has the highest expression levels in the kidneys of mammals, which is additionally upregulated in diabetic-induced CKD, indicating that DPP-4 inhibition by sitagliptin is a plausible therapeutic target for management of DN. In fact, several studies have been describing putative pleiotropic effects of sitagliptin on various organs and tissues. Sitagliptin showed not only the capacity to ameliorate diabetic dysmetabolism but also the potential to avert the decline of insulin secretion ability in pancreatic beta-cells through cytoprotective properties; these effects suggest a role in prevention of T2DM evolution and its complications. In the kidney, sitagliptin seems to provide renoprotection by restoring GLP-1 diuretic and natriuretic actions and by other mechanisms, including antiapoptotic, antifibrotic, anti-inflammatory, and antioxidant effects. However, additional studies are needed to clarify whether sitagliptin acts through indirect action via insulin secretion increment or through direct tissular DPP-4 inhibition. In addition, further research should also elucidate the contribution of GLP-1-dependent (which is sustained by expression of DPP-4 and GLP-1R in renal tissues) and/or GLP-1-independent pathways (reinforced by the existence of multiple DPP-4 substrates).

Due to its unique mechanism of action and pharmacological properties, DPP-4 inhibitors (including sitagliptin) have conquered their place in T2DM management. In addition, the low potential for interactions with other antidiabetic drugs allows its use in different combinations, with a low risk of hypoglycaemiac episodes. A fixed-dose combination with sodium/glucose cotransporter 2 (SLGT2) inhibitor ertugliflozin has been recently accepted and seems to contain the potential to exert further beneficial effects on the kidney, as both classes have been reported to lower UAER. Additional positive effects could be expected from the complementary mechanism of action of these drugs, with impact on both renal and cardiovascular systems. Disclosure of its protective actions on the diabetic kidney could open up the possibility of using sitagliptin therapy as a renoprotective strategy against the development and/or delay of DN.
